# Supplementary material for: Strange attractor of a narwhal (Monodon monoceros)
Source: PLoS Comput Biol. 2022 Sep 22;18(9):e1010432. doi: 10.1371/journal.pcbi.1010432 (PMC9498936; doi:10.1371/journal.pcbi.1010432)
Supplement: S1 Appendix — (PDF) [file pcbi.1010432.s001.pdf]

S1 Appendix

Supporting Figures

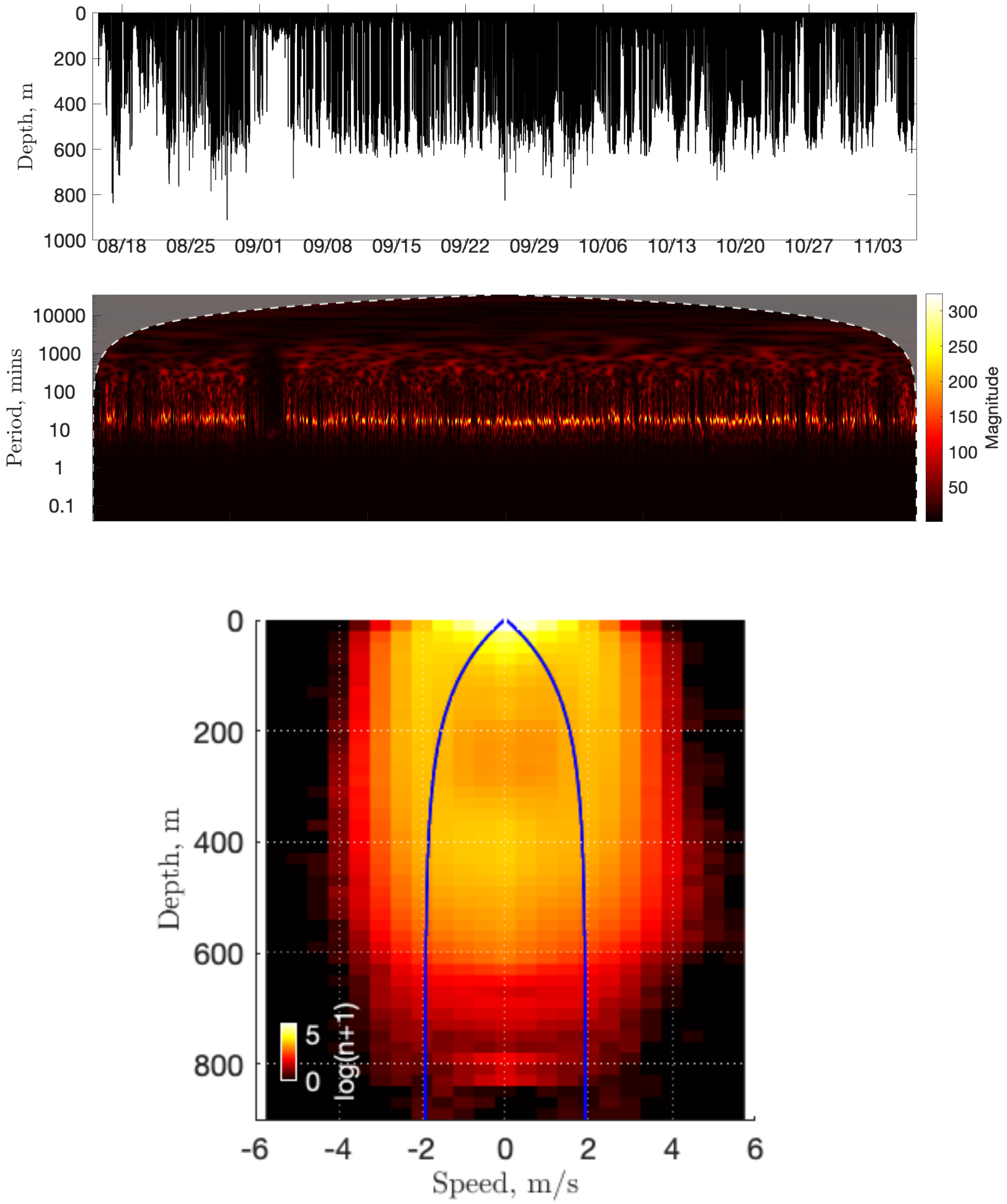

Fig A. Dive records including period and speed.

Narwhal depth readings used in the study (August 15 to November 6, 2013; Summer East Greenland Time or UTC) with the corresponding continuous 1D wavelet transform (CWT) of data (the cone of influence is shown in grey; the dominant time scale is ~17 min), and the rate of depth change with time (i.e., vertical speed) versus depth. The density map was computed using 20 m depth bins and 0.5 m/s speed bins and taking a $\log_{10}$ of (n+1) to compensate for the large number of points near the surface. Blue curves reflect the empirical equation for mean vertical speed to destination depth for 36 narwhals in Baffin Bay [1].

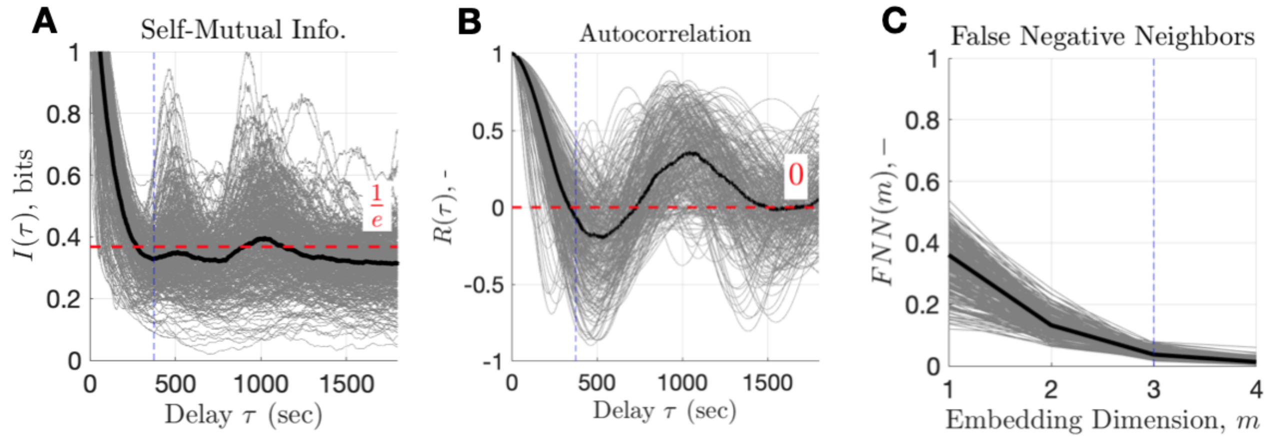

Fig B. Embedding parameters.

(A) Self-Mutual Information and **(B)** autocorrelation as functions of a time delay, τ. Dashed vertical lines indicate the chosen delay for embedding (τ = 375 s). **(C)** False Negative Neighbours as a function of embedding dimension (τ = 375 s, r = 3). Dashed vertical line corresponds to the chosen m = 3. Each grey curve corresponds to computation for a 6 h time segment of the record; think black curve shows the median estimate.

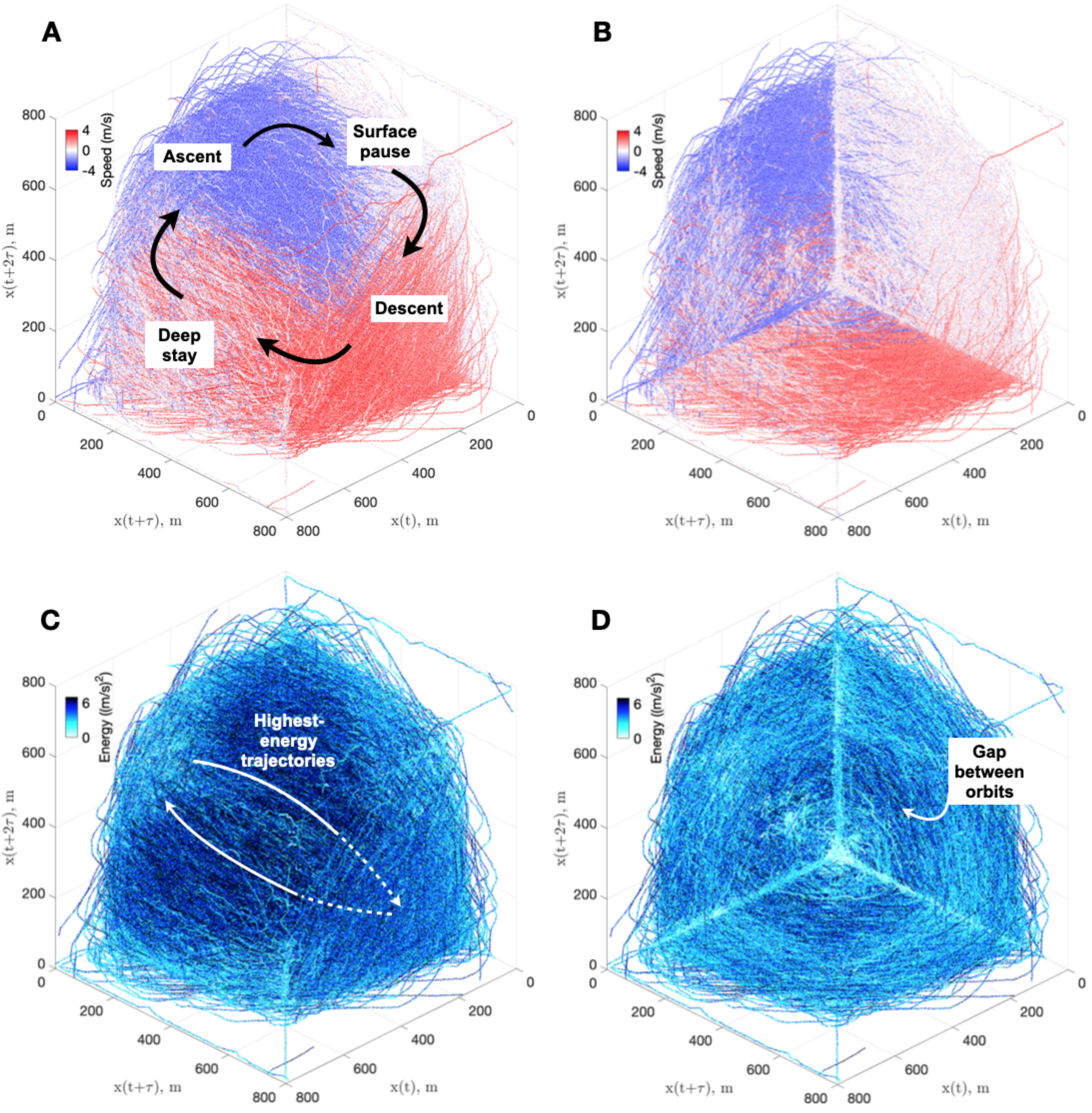

Fig C. Trajectory epochs and energetics.

(A) Points of behavior space colored by the associated speed (i.e., dD/dt). Different epochs tend to form clockwise flow via regions corresponding to (1) a short pause before the next dive of the bout (white), (2) descent (red), (3) deceleration and stay at depth (again white), and (4) ascent back to the surface (blue). **(B)** Plot showing the same scatter as in (A) but with semi-transparent colors to highlight the tendency of the three dominant epochs of short pause, descent, and ascent to stay on the three main planes. **(C)** Instantaneous kinetic energy of points along trajectories, showing the most energetic paths. **(D)** Plot showing the same scatter as in (C), but with semi-transparent color to highlight that the core and pathways to it have reduced energy. The gap between the inner and outer orbitals can be also seen.

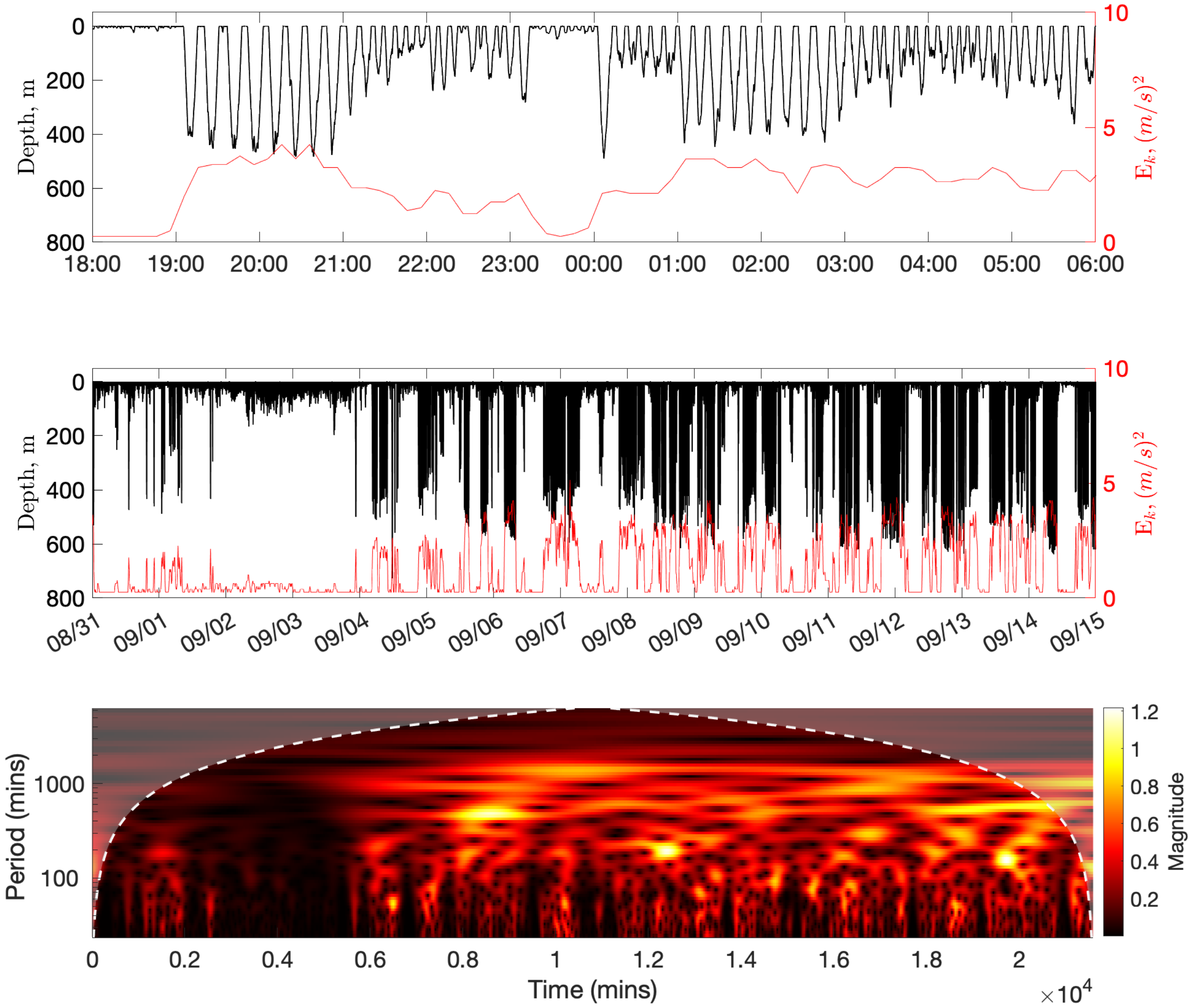

Fig D. Examples of kinetic energy variation.

Kinetic energy, E_m, and depth variation between 18:00 September 24 and 06:00 September 25, 2013 (upper subplot) and August 31 to September 15, 2013 (lower subplot). For the latter time interval, the corresponding two-week CWT is also shown.

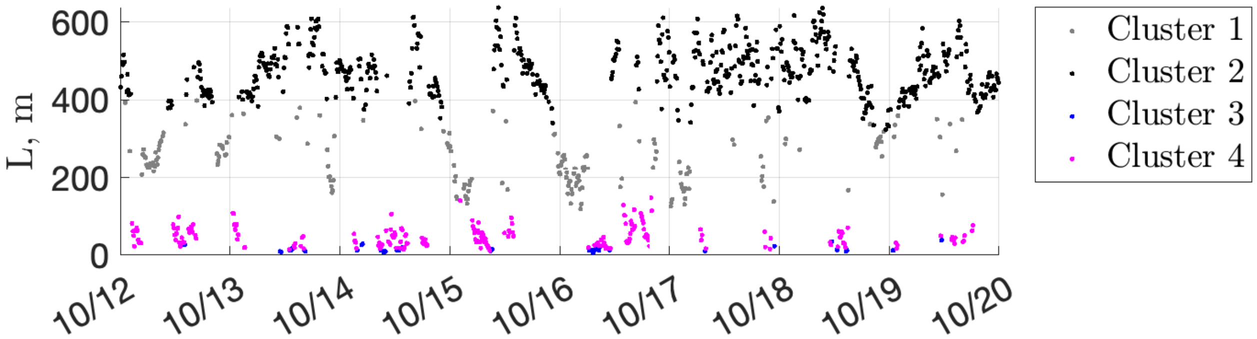

Fig E. Near-midnight transitions in decoded Euclidian distances.

Example of de-clustered L (October 12–20, 2013) illustrating smooth deep-to-shallow transitions. Only points with the highest membership scores (>0.9) are shown.

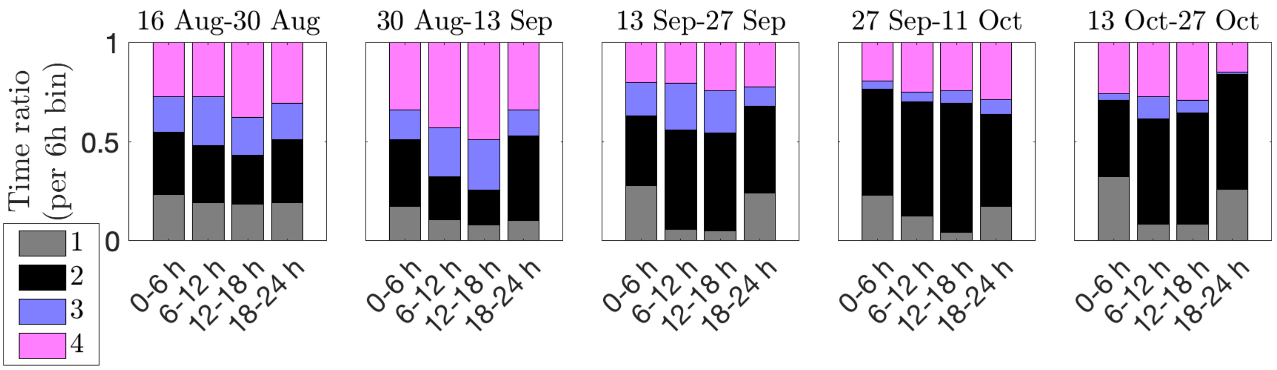

Fig F. Shift in dedication to different types of activity.

Long-term variation of relative time partitioning of activities (i.e., clusters) by time of a day, using 6 h bins and two-week time segments. Marginal data (for August 15, 2013 and after October 27, 2013) were omitted for consistent binning.

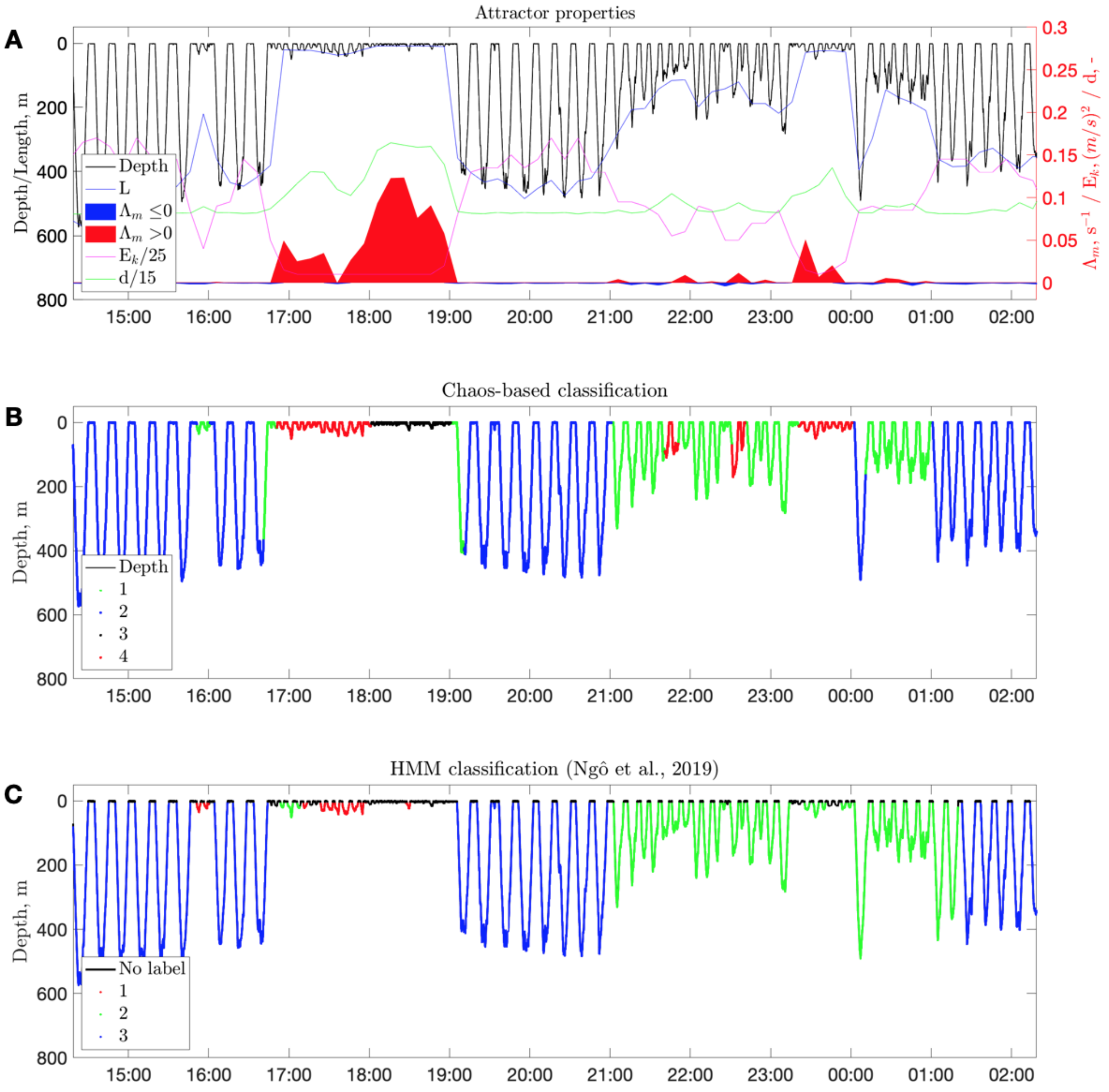

Fig G. From attractor properties to diving behavior classification.

(A) Properties of the attractor used for classification (12 h of record from September 24, 2013 14:18:39). **(B)** Corresponding diving behavior state decoding based on chaotic measures clustering (continuous). **(C)** The estimated state (per dive) using HMMs (data from Fig. 8 of [2], where date-stamp was offset by –48 h. Note that time intervals between dives of the same bout type are not assigned to the bout by HMMs in (C) ("No label"), contrary to assignment by chaos-based classifier in (B).

Supporting Movie and Data files

Video A. Available at [3; https://zenodo.org/record/6522945#.YsUHnuyRWX1] as DepthNarwhal_raw_CWT_2minstep_LowRes.mp4 (292 Mb)

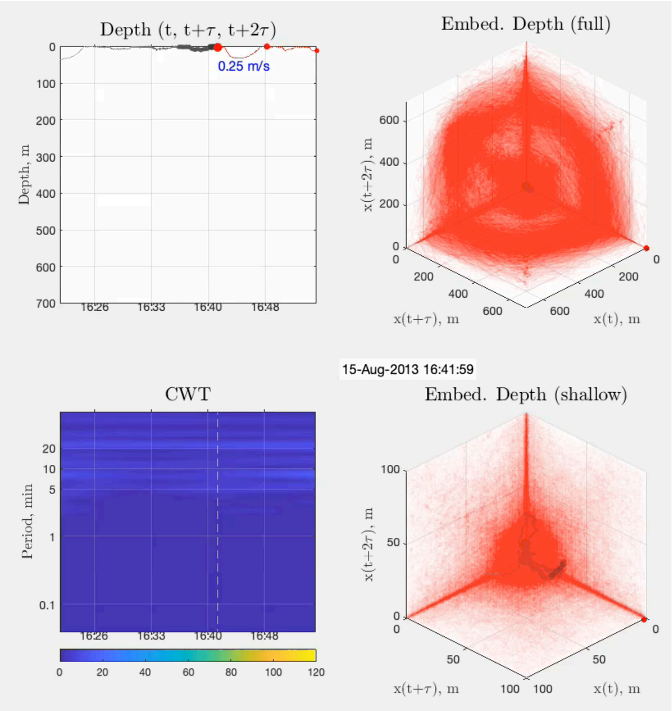

Animation of original depth readings and corresponding continuous wavelet transforms, CWTs, versus time-delay embedding reconstruction (m = 3, τ = 375 s). Upper-left panel shows the raw data, where the largest red circle indicates the current time step, $x(t_i)$ (with the associated rate of depth change, in m/s), and two smaller red circles the corresponding $x(t_i + \tau)$ and $x(t_i + 2\tau)$. The tail with decreasing thickness in black corresponds to the final 20 min of data. Lower-left panel shows continuous wavelet transform (CWT) of the data with magnitude by color. The corresponding space–state reconstruction is presented to the right, where the lower-right panel is enlarged for better display of the "core". The tail of the preceding 20 min of data is shown in black to aid visual tracking of recent history. The red transparent background is the full embedding result. Video is 3,609 times faster than reality; frame time-step is 2 min.

Video B. Available at [3; https://zenodo.org/record/6522945#.YsUHnuyRWX1] as attractor_6h_steps.mp4 (8 Mb).

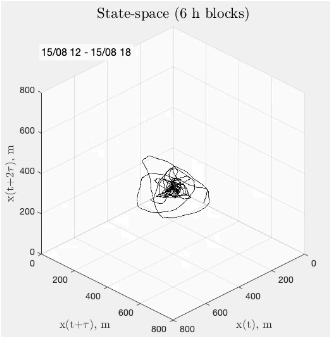

State–space reconstruction animated using 6 h time-steps for highlighting long-term variability of narwhal diving behavior.

File A. Available at [3; https://zenodo.org/record/6522945#.YsUHnuyRWX1] as Whale3965nodry.csv (143 Mb)

Depth readings in m, at 1 sample per second (2013) (for Fig A in S1 Appendix).

References

1. Laidre KL, Heide-Jørgensen MP, Dietz R, Hobbs RC, Jørgensen OA, Deep-diving by narwhals monodon monoceros: differences in foraging behavior between wintering areas? Mar. Ecol. Prog. Ser. 2003;261: 269-281.

2. Ngô MC, Heide-Jørgensen MP, Ditlevsen S, Understanding narwhal diving behaviour using Hidden Markov Models with dependent state distributions and long range dependence. PLOS Comput. Biol. 2019;15: 1-21.

3. Podolskiy EA, Heide-Jørgensen MP, Nonlinear analysis of narwhal dive records (data and animation). Zenodo. 2022. doi: 10.5281/zenodo.6522945. https://zenodo.org/record/6522945#.YsUHnuyRWX1
