# Supplementary material for: Strange attractor of a narwhal (Monodon monoceros)
Source: PLoS Comput Biol. 2022 Sep 22;18(9):e1010432. doi: 10.1371/journal.pcbi.1010432 (PMC9498936; doi:10.1371/journal.pcbi.1010432)
Supplement: S1 Fig — Violin plots of dimensionality, d, by hour of day (using 3 h bins) with red and blue curves connecting percentile levels (P50% and/or P5,75%). In the center column, the corresponding percentile levels are shown on a circular 24 h plot (representing the narwhal daily routine; yellow circle indicates the sun’s culmination time, 13:29; arrows indicate the time of sunset and sunrise on September 25, 2013 the middle date of the dataset). Right side: empirical Cumulative Distribution Function (eCDF), for the same quantities computed using 6 h bins (black represents “night”, red “day”). (PDF) [file pcbi.1010432.s002.pdf]

S1 Fig

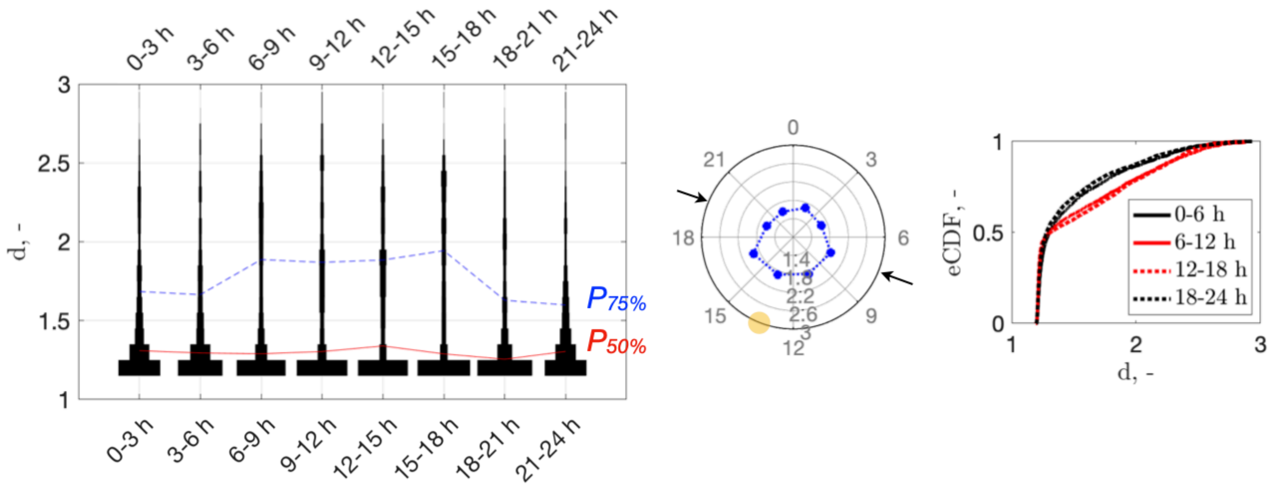

S1 Fig. Diurnal narwhal behavior (dimensionality).

Violin plots of dimensionality, d, distributions by hour of day (using 3 h bins) with red and blue curves connecting percentile levels ($P_{50\%}$ and/or $P_{5,75\%}$). In the center column, the corresponding percentile levels are shown on a circular 24 h plot (representing the narwhal daily routine; yellow circle indicates the sun’s culmination time, 13:29; arrows indicate the time of sunset and sunrise on September 25, 2013 the middle date of the dataset). Right side: empirical Cumulative Distribution Function (eCDF), for the same quantities computed using 6 h bins (black represents “night”, red “day”).
