# Supplementary material for: Strange attractor of a narwhal (Monodon monoceros)
Source: PLoS Comput Biol. 2022 Sep 22;18(9):e1010432. doi: 10.1371/journal.pcbi.1010432 (PMC9498936; doi:10.1371/journal.pcbi.1010432)
Supplement: Attachment — Submitted filename: Reviewer report on manuscript PCOMPBIOL-D-22-00691.pdf [file pcbi.1010432.s003.pdf]

Reviewer report on: "Strange attractor of a narwhal (Monodon monoceros)" (PCOMPBIOL-D-22-00691)

June 24, 2022

In their manuscript entitled "Strange attractor of a narwhal (Monodon monoceros)" (PCOMPBIOL-D-22-00691), the authors propose to use chaos theory to detect structures in the diving behaviour of a narwhal. I enjoyed reading the manuscript and find the suggested approach both innovative and potentially useful. The provided supplementary material nicely complements the manuscript; I particularly enjoyed watching the videos, which help to understand the plots displayed in the manuscript.

Some comments on the manuscript, questions, and suggestions for improvements are provided below.

1. I believe that the manuscript could be improved by making it more accessible to readers who are not familiar with chaos theory. For instance, is there any definition for chaos (i.e., what does the term mean in the context of chaos theory)? This could be explained around line 15. Also, the term time-delayed embedding could be explained in more detail around line 17.

2. Related to the above item, I suggest to briefly explain some terms related to chaos theory (either in the main body of the text or in a box) so that readers without a background in chaos theory can better follow the manuscript. There are some useful explanations in the supplementary material (e.g., on the Lyapunov exponent), however, I believe that it would be useful if those terms were explained in a sentence or so in the main body of the text.

3. In line 25, you mention hidden Markov models (HMMs). I suggest to highlight the differences between your method and HMMs in a bit more detail. From my understanding, the main difference is that your method is merely data-driven, whereas HMMs constitute a probabilistic modelling approach (which has both advantages and disadvantages; e.g., your method may be better suited as an exploratory tool, whereas HMMs may be better suited to explicitly model switches between states).

4. Related to the above item, in line 170, you mention that it is not necessary to choose the number of states a-priori. I believe that this is an important advantage that could be highlighted a bit earlier in the manuscript. Do you believe that your method can also be useful for selecting the number of states of an HMM (if you are interested in probabilistic modelling), or would you not recommend this, as there is no one-to-one correspondence between the states inferred using your method and those inferred by HMMs?

5. The suggested approach relies on a visual assessment of the results. As you analysed data collected on a single individual, I was wondering how useful it would be for larger data sets. In particular, would it be possible to infer and compare behaviours from multiple individuals, species, etc. (I am sure that the answer to that question would be yes, as noted around line 217, but perhaps you can comment in a bit more detail on the challenges)?

6. I was wondering what conditions the data to be analysed have to satisfy such that your method is able to detect states? Specifically, is it also applicable to tracking data from other species, such as birds or terrestrial animals, or is the special structure of marine mammal data crucial for your method? I suggest to briefly discuss potential applications beyond marine mammal data.
