# Supplementary material for: Strange attractor of a narwhal (Monodon monoceros)
Source: PLoS Comput Biol. 2022 Sep 22;18(9):e1010432. doi: 10.1371/journal.pcbi.1010432 (PMC9498936; doi:10.1371/journal.pcbi.1010432)
Supplement: Attachment — Submitted filename: rebuttal_letter_PLOS_3Jul2022.pdf [file pcbi.1010432.s005.pdf]

July 3, 2022

We thank the editors and reviewers for evaluating our submission and encouraging us to improve the manuscript. We appreciate their time and thoughtful comments. The revised manuscript is enclosed, and our detailed point-by-point responses are given below (shown in **bold**; modifications are highlighted red in the manuscript). We believe these modifications have strengthened the paper.

Comments from the Reviewers:
****
Reviewer 1:

In their manuscript entitled “Strange attractor of a narwhal (Monodon monoceros)” (PCOMPBIOL-D-22-00691), the authors propose to use chaos theory to detect structures in the diving behaviour of a narwhal. I enjoyed reading the manuscript and find the suggested approach both innovative and potentially useful. The provided supplementary material nicely complements the manuscript; I particularly enjoyed watching the videos, which help to understand the plots displayed in the manuscript.

Thank you very much!

Some comments on the manuscript, questions, and suggestions for improvements are provided below.
1. I believe that the manuscript could be improved by making it more accessible to readers who are not familiar with chaos theory. For instance, is there any definition for chaos (i.e., what does the term mean in the context of chaos theory)? This could be explained around line 15. Also, the term time-delayed embedding could be explained in more detail around line 17.

As suggested, in the Introduction, the terms of chaos, embedding, and attractor have been explained.

2. Related to the above item, I suggest to briefly explain some terms related to chaos theory (either in the main body of the text or in a box) so that readers without a background in chaos theory can better follow the manuscript. There are some useful explanations in the supplementary material (e.g., on the Lyapunov exponent), however, I believe that it would be useful if those terms were explained in a sentence or so in the main body of the text.

Additionally to corrections regarding the previous comment, this was implemented in the main text where appropriate (for Euclidean distance, exponent, energy, etc.).

3. In line 25, you mention hidden Markov models (HMMs). I suggest to highlight the differences between your method and HMMs in a bit more detail. From my understanding, the main difference is that your method is merely data-driven, whereas HMMs constitute a probabilistic modelling approach (which has both advantages and disadvantages; e.g., your method may be better suited as an exploratory tool, whereas HMMs may be better suited to explicitly model switches between states).

Addressed. Please, see below.

4. Related to the above item, in line 170, you mention that it is not necessary to choose the number of states a-priori. I believe that this is an important advantage that could be highlighted a bit earlier in the manuscript. Do you believe that your method can also be useful for selecting the number of states of an HMM (if you are interested in probabilistic modelling), or would you not recommend this, as there is no one-to-one correspondence between the states inferred using your method and those inferred by HMMs?

These 2 thoughtful comments give us pause. After careful consideration, in order to keep our claims, first, not overhyped, and two, within the scope of presented materials, we included the following paragraph to the Introduction: “In this first attempt to compare such dissimilar conceptual toolkits, it is premature to identify the main difference. We conjecture that a time-delay embedding is a data-driven method suited as an exploratory tool. In contrast, HMMs correspond to a probabilistic modelling approach suited to model switches between states. Moreover, even if there is no one-to-one correspondence between the states identified using both methods, we presume that our method might help in selecting the number of states of HMMs. Thus, we advocate for future experiments to confirm this potential advantage.”

5. The suggested approach relies on a visual assessment of the results. As you analysed data collected on a single individual, I was wondering how useful it would be for larger data sets. In particular, would it be possible to infer and

compare behaviours from multiple individuals, species, etc. (I am sure that the answer to that question would be yes, as noted around line 217, but perhaps you can comment in a bit more detail on the challenges)?

Our answer is yes, indeed. This is a great point to elaborate on. So we mentioned in the Discussion that, actually, the theory of nonlinear dynamical systems offers many tools for the diagnosis of synchronization, coupling and uncovering variation among individuals.

6. I was wondering what conditions the data to be analysed have to satisfy such that your method is able to detect states? Specifically, is it also applicable to tracking data from other species, such as birds or terrestrial animals, or is the special structure of marine mammal data crucial for your method? I suggest to briefly discuss potential applications beyond marine mammal data.

This is a good question. We think that, in practice, a recurrence of behaviour and sufficient sampling are the main requirements to the data. Thus the approach is broadly applicable, as has been illustrated by our references on other species (flies, humans, cows). We briefly discussed this aspect in a new paragraph to the Discussion.

****
Reviewer 2:

The authors in this study present a novel computational approach to quantifying the diving behaviors of a narwhal. They have applied tools from chaos theory to analyze the 1D time-series depth data obtained experimentally by tagging a narwhal, and successfully quantify the different behaviors – such as activity types, diurnal patterns and seasonal trends. Here, they build upon their previous Hidden Markov Model (HMM) analysis of the same experimental dataset (Ngo et al, PLOS Comput. Biol. 2019) and demonstrate the advantages of using this chaos theory approach. I am impressed with all the different characteristics that they are able to quantify using a single time-series measurement. I strongly believe that this manuscript would be a very useful tool to quantify so many different types of animal behavior and is a welcome addition for the broad community of biologists, physicists, and probably mathematicians too. In the coming years, I am excitedly looking forward to the extension of this study to cover the behaviors of a population of animals and their communities. For all these compelling reasons, I recommend publication of this manuscript in PLOS Computational Biology.

Thank you very much for your careful reading and such encouraging feedback!

I have a few minor requests/clarifications for the authors to take into consideration before publication of this manuscript:
1) Code sharing: It would be great if the authors make an ‘open source’ version of their code available for the community. This would encourage others to use and build upon this study.

The code is shared at https://github.com/Jehiko/narwhal/

2) Acceleration: Have the authors looked into whether acceleration could be useful as an epoch indicator? It would be useful to comment on this, even if it is a potential item for future study.

We agree that acceleration could be informative, and some previous studies relied on it [Frank et al., 2011; Sturm et al. 2019]. For our experiment, we did not use accelerometers, but hope to work with such data in the future. In regard to derivatives, we explained that using (x, x’, x’’) yields topologically similar attractor [Packard et al., 1980]. We explained this in the Materials and Methods section.

3) 3-D whale trajectories: Can the authors describe in much more detail (tutorial style) how the iterated mapping allows reconstruction of 3-D trajectories, from 1-D measurements?

In the Introduction, we provided more technical background to clarify this procedure.

****
Once again, we thank the Reviewers for their help to improve our manuscript.
