# Supplementary material for: Strange attractor of a narwhal (Monodon monoceros)
Source: PLoS Comput Biol. 2022 Sep 22;18(9):e1010432. doi: 10.1371/journal.pcbi.1010432 (PMC9498936; doi:10.1371/journal.pcbi.1010432)
Supplement: Attachment — Submitted filename: Podolsky_narwhal_PlosCompBio_Report.docx [file pcbi.1010432.s004.docx]

**Report on Manuscript “Strange attractor of a narwhal (*Monodon monoceros*)”**

**By E. A. Podolsky and M. P. Heide-Jorgensen**

The authors in this study present a novel computational approach to quantifying the diving behaviors of a narwhal. They have applied tools from chaos theory to analyze the 1D time-series depth data obtained experimentally by tagging a narwhal, and successfully quantify the different behaviors – such as activity types, diurnal patterns and seasonal trends. Here, they build upon their previous Hidden Markov Model (HMM) analysis of the same experimental dataset (Ngo et al, PLOS Comput. Biol. 2019) and demonstrate the advantages of using this chaos theory approach. I am impressed with all the different characteristics that they are able to quantify using a single time-series measurement. I strongly believe that this manuscript would be a very useful tool to quantify so many different types of animal behavior and is a welcome addition for the broad community of biologists, physicists, and probably mathematicians too. In the coming years, I am excitedly looking forward to the extension of this study to cover the behaviors of a population of animals and their communities. For all these compelling reasons, I recommend publication of this manuscript in PLOS Computational Biology.

I have a few minor requests/clarifications for the authors to take into consideration before publication of this manuscript:

1) Code sharing: It would be great if the authors make an ‘open source’ version of their code available for the community. This would encourage others to use and build upon this study.

2) Acceleration: Have the authors looked into whether acceleration could be useful as an epoch indicator? It would be useful to comment on this, even if it is a potential item for future study.
3) 3-D whale trajectories: Can the authors describe in much more detail (tutorial style) how the iterated mapping allows reconstruction of 3-D trajectories, from 1-D measurements?
